# Study on the Changes of Brain Function in Adolescents With Pain‐Depression Comorbidity Based on rs‐FMRI

**DOI:** 10.1155/da/7986150

**Published:** 2025-12-11

**Authors:** Yu Tian, Wenyu Cai, Changjing He, Gaoqiang Xu, Ganjun Song, Kuntao Chen, Songjiang Liu

**Affiliations:** ^1^ Department of Radiology, The Affiliated Hospital of Zunyi Medical University, Zunyi, China, zmchospital.com.cn; ^2^ Chongqing Tongliang District People’s Hospital, Chongqing, China; ^3^ Department of Radiology, The Fifth Affiliated Hospital of Zunyi Medical University (Zhuhai), Zhuhai, China, zmc.edu.cn

**Keywords:** adolescent, comorbidity, depression, pain, resting state functional magnetic resonance imaging

## Abstract

**Background:**

The underlying mechanism of pain‐depression comorbidity is not well understood. This study aims to analyze the abnormal brain activity in adolescents with pain‐depression comorbidity, and to provide imaging evidence for the understanding of brain neural mechanisms of pain‐depression comorbidity.

**Methods:**

Depression in adolescents with (*n* = 31) and without (*n* = 26) comorbid pain symptoms and 28 healthy controls matched for age, gender, and education level underwent resting‐state functional magnetic resonance imaging (rs‐fMRI) and completed the Hamilton depression scale and visual analog scale (VAS) of pain. The whole‐brain amplitude of low‐frequency fluctuation (ALFF) and regional homogeneity (ReHo) values were compared across groups. The brain regions with significant differences in ALFF values between groups were used as seed points for whole‐brain functional connectivity.

**Results:**

Compared with the depression group, the comorbidity group showed increased ALFF values in the right amygdala and right middle frontal gyrus, decreased ALFF values in the left middle occipital gyrus, right inferior temporal gyrus and right superior temporal gyrus, increased ReHo values in the right insula, and elevated functional connectivity between the right inferior temporal gyrus and right angular gyrus (Gaussian random field [GRF] corrected, *p*  < 0.05).

**Conclusion:**

Compared with adolescents with depression without pain, adolescents with pain‐depression comorbidity have differences in neuronal activity and functional connectivity in the middle frontal gyrus, amygdala, insular lobe, temporoparietal and occipital lobes, suggesting that abnormal neuronal activity in these brain regions may be the neural basis of pain‐depression comorbidity.

## 1. Introduction

Depression is a common mental disorder characterized by persistent low mood and reduced volitional activity, and is a prevalent mental illness during childhood and adolescence. Epidemiological surveys indicate that adolescent depression is characterized by high incidence, suicide rates, and recurrence rates, with a lifetime prevalence of ~11%. Nearly 30% of adolescents with severe depression have suicidal tendencies [1, 2]. In addition to common mood symptoms, depression patients often experience various somatic pains, such as back pain, headaches, abdominal pain, and chest pain. These pain symptoms significantly impact patients’ quality of life and are common causes of disability. Studies estimate that 20%–35% of children and adolescents worldwide are affected by pain [3, 4]. Pain and depression interact, exacerbating each other, making the combination more disabling and costly than either condition alone. This interaction can also lead to treatment‐resistant conditions, making patients less responsive to medication. The intertwined clinical features of pain and depression suggest a possible shared pathogenesis. Antidepressants, such as serotonin‐norepinephrine reuptake inhibitors, have been widely proven and used in clinical pain treatment. Additionally, the timing of the antidepressant effects and analgesic effects on chronic pain differs, indicating that pain and depression may involve different mechanisms [5]. Functional magnetic resonance imaging (fMRI), as an objective and noninvasive neuroimaging measurement method, can provide insights into brain function changes in adolescents with comorbid pain and depression. This is crucial for further exploring the neural mechanisms related to pain processing in depression patients.

Current hypotheses on the mechanisms of comorbidity include neurotransmitter dysfunction, inflammatory factors, and reduced neuroplasticity [6–8]. These primarily lead to neuronal damage in brain regions related to emotion and pain, such as the frontal cortex, hippocampus, and amygdala, ultimately resulting in depressive and pain symptoms. However, the neural mechanisms underlying the comorbidity of pain and depression are not fully understood. Resting‐state functional magnetic resonance imaging (rs‐fMRI) technology indirectly reflects the activation levels of neurons in various brain regions and their intrinsic connections by detecting blood oxygen levels in brain tissue. This technique is now widely used in the study of various mental illnesses, including depression. Main research methods include amplitude of low‐frequency fluctuation (ALFF), regional homogeneity (ReHo), and functional connectivity. ALFF describes the fluctuation characteristics of BOLD signals during neural activity within brain regions, reflecting changes in neuronal activation during the resting state. ReHo measures the synchronization of BOLD signal activity between adjacent voxels, assessing the consistency of local brain neuronal activity. Functional connectivity is defined as the temporal dependance of neuronal activity patterns in anatomically separated brain regions, reflecting functional communication between brain regions by measuring the co‐activation levels of fMRI time series [9].

Previous studies using pain stimuli to investigate brain activation changes in depression patients found increased activation in the prefrontal cortex and postcentral gyrus [10, 11], while functional connectivity in the frontal, temporal, and occipital neural networks was significantly reduced [12]. Zhang et al. [13] found abnormal functional connectivity in the left medial prefrontal cortex and multiple parietal cortices in patients with comorbid depression and chronic low back pain. Additionally, topological properties such as small‐world attributes, local efficiency, and normalized clustering coefficients were also abnormal, indicating that depressive symptoms exacerbate functional changes in the pain matrix of chronic low back pain patients. In a rat model of comorbid chronic pain and depression, asymmetric activation of the locus coeruleus [14] and hippocampus [15] played an important role in regulating the comorbidity of pain and depression, suggesting that neuroplastic changes in noradrenergic neurons of the locus coeruleus and microglia in the hippocampus may be one of the neural mechanisms underlying the comorbidity of pain and depression. A recent study also revealed that enhanced functional projection from the lateral habenula to the ventral tegmental area may be key to chronic pain‐induced depression [16].

Previous neuroimaging studies based on fMRI technology have found abnormal brain functional activities in patients with comorbid pain and depression, as well as in depression patients, yielding significant results. These abnormal brain regions include the prefrontal cortex, amygdala, anterior cingulate cortex, hippocampus, thalamus, and insula [17, 18]. However, most comorbidity studies construct comorbidity models by experimentally inducing pain or depressive symptoms, which are temporary and do not fully simulate the real clinical situation of comorbid patients. Moreover, there are few studies on the application of rs‐fMRI in adolescent patients with comorbid pain and depression. Therefore, this study aims to use fMRI technology to compare and analyze differences in local brain region ALFF, ReHo, and functional connectivity changes between adolescent patients with comorbid pain and depression and depression patients during the resting state. This will explore the neural activity characteristics of adolescent patients with comorbid pain and depression, providing imaging evidence for understanding the neural mechanisms of pain‐depression comorbidity. This methodology has also been applied to investigate alterations in brain function among patients with residual dizziness caused by benign paroxysmal positional vertigo [19].

## 2. Materials and Methods

### 2.1. Participants

This study used one‐way analysis of variance, two‐sided test, effect size *f* = 0.4, *α* = 0.05, power *p* = 0.95, number of groups *n* = 3, the total sample size calculated by Gpower3.1 was 102, and the average sample size of each group was 34. This study enrolled 40 first‐episode drug‐naive patients with comorbid depression and 30 patients with depression from the Affiliated Hospital of Zunyi Medical University, and recruited 32 healthy volunteers from the local community. All subjects were diagnosed by a psychiatrist with the title of attending physician or higher according to the Diagnostic and Statistical Manual of Mental Disorders, Fifth Edition (DSM‐5). The inclusion and exclusion criteria are as follows:

#### 2.1.1. Inclusion and Exclusion Criteria for the Pain‐Depression Comorbidity Group

The inclusion criteria were as follows: (1) meets DSM‐5 diagnostic criteria, HAMD ≥ 7, VAS＞0, (2) aged 12–18, right‐handed, (3) no history of severe head trauma or other mental illnesses, (4) no contraindications for MRI, (5) no prior psychological or drug/physical therapy. The exclusion criteria were as follows: (1) inability to complete fMRI scanning, (2) abnormal findings on routine MRI of the brain, and (3) MRI image quality did not meet data processing requirements.

#### 2.1.2. Inclusion and Exclusion Criteria for the Depression Group

The inclusion criteria are the same as those for the pain‐depression comorbidity group, except for the first criterion: meets DSM‐5 diagnostic criteria, HAMD ≥ 7, VAS = 0.

#### 2.1.3. Inclusion and Exclusion Criteria for the Control Group

The inclusion criteria were as follows: (1) healthy volunteers matched for age, gender, handedness, and education level with the case groups, (2) no history of severe head trauma or other mental illnesses, (3) no contraindications for MRI, (4) no prior psychological or drug/physical therapy. The exclusion criteria were as follows: (1) HAMD ≥ 7, VAS > 0, (2) inability to complete fMRI scanning, (3) abnormal findings on routine MRI of the brain, and (4) MRI image quality does not meet data processing requirements.

Furthermore, ethics approval for this study was granted by the Ethics Committee of the Affiliated Hospital of Zunyi Medical University, code number KLLY‐2022‐189. All steps involving human subjects adhered to the Declaration of Helsinki. The content of this research paper was solely completed by the authors and co‐authors without the use of AI‐assisted tools for content generation. Finally, parents or guardians of each participant provided written informed consent to participate in the research.

### 2.2. Data Acquisition

#### 2.2.1. General Data Collection

Before the MRI examination, clinical data such as gender, age, and education level were collected. The Hamilton depression rating scale (HAMD) and visual analog scale (VAS) were used to assess the severity of depression and pain in the subjects.

#### 2.2.2. MRI Data Acquisition

All imaging data were collected using A GE Signa HDxt 3.0T MR scanner (GE Healthcare, Milwaukee, Wisconsin, USA) at the Department of Radiology at the Affiliated Hospital of Zunyi Medical University. During scanning, the participants were instructed to lie still, close their eyes, and relax. In addition, a three‐dimensional brain volume sequence (3D BRAVO) was applied to obtain structural T1‐weighted images (repetition time [TR]: 7.8 ms; echo time [TE]: 3.0 ms; slice thickness: 1.0 mm; field of view (FOV), 256 mm × 256 mm; matrix, 256 × 256; flip angle: 15°). Moreover, functional images were acquired using the gradient‐recalled echo‐planar imaging (EPI) sequence (TR, 2000 ms; TE: 30 ms; flip angle, 90°; slice thickness: 4.0 mm; slice gap, 0 mm; FOV, 240 mm × 240 mm; matrix, 256 × 256).

### 2.3. Data Preprocessing

DPABI (http://rfmri.org/dpabi) and SPM12 (http://www.fil.ion.ucl.ac.uk/spm) software packages were used to preprocess and analyze ALFF and ReHo values from the MRI data of the three groups. Brain regions with ALFF differences between the pain‐depression comorbidity group and the depression group were used as seed points for whole‐brain functional connectivity analysis of preprocessed rs‐fMRI data. This procedure included: (1) data format conversion: Converting the DICOM image to NIFTI format; (2) remove the first 10‐time series images; (3) slice timing correction; (4) head movement correction; (5) covariate regression; (6) normalizing to the Montreal Neurologic Institute (MNI) space and resampling; (7) linear de‐trending, and filtering (0.01–0.1 Hz); (8) based on SPM8 software, the ALFF value of each subject was smoothed by a 4‐mm full‐width at half‐maximum (FWHM) kernel; (9) functional connectivity analysis: the brain regions with ALFF differences between the pain‐depression comorbidity group and the depression group were used as seed points, and the whole brain functional connectivity analysis was performed on the preprocessed rs‐fMRI data.

### 2.4. Statistical Analysis

General clinical data: SPSS 29.0 (IBM Corp, Armonk, NY, USA) was used for statistical analysis of clinical data from the three groups. First, the normal distribution and homogeneity of variance of age, education years, and HAMD scores were tested. One‐way ANOVA or Kruskal–Wallis H test was used for continuous variables, and the chi‐square test was used for gender. The significance level was set as *p* < 0.05.

Imaging data: Differences in whole‐brain ALFF, ReHo, and functional connectivity among the three groups were analyzed using the rs‐fMRI data analysis toolkit (DPABI) with one‐way ANCOVA, using age, gender, education level, and motion parameters (Mean FD) as covariates. Post‐hoc comparisons were corrected using the Bonferroni method, and the significance threshold was corrected based on the Gaussian random field (GRF) method, with a voxel‐level *p*  < 0.005 and cluster‐level *p*  < 0.05 considered statistically significant.

Correlation analysis: SPSS 29.0 was used to perform Pearson correlation analysis between ReHo, ALFF, and functional connectivity values of brain regions with differences among the three groups and HAMD, VAS scores. The significance level was set as *p* < 0.05.

## 3. Results

### 3.1. Demographic Characteristics

**Figure 1:**
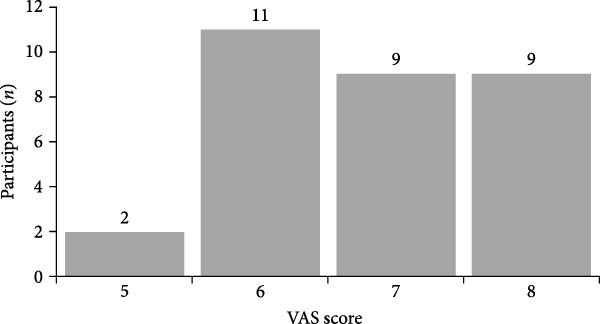
VAS scores in the comorbidity group.

This study collected data from 40 adolescents diagnosed with comorbid pain and depression (referred to as the comorbidity group), 30 adolescents with depression without pain (referred to as the depression group), and 32 healthy volunteers matched for age, gender, and education level (referred to as the control group) at the Affiliated Hospital of Zunyi Medical University. After screening based on inclusion and exclusion criteria, 31 subjects in the comorbidity group, 26 in the depression group, and 28 in the control group were included in the study. General data of the subjects are shown in Table 1, and the VAS scores of the comorbidity group are shown in Figure 1. There were no significant differences in age, gender, education level, or motion parameters among the groups (*p* > 0.05). HAMD scores in the comorbidity and depression groups were higher than those in the healthy control group, with significant differences (*p* < 0.05), but there was no significant difference in HAMD scores between the comorbidity and depression groups (*p* > 0.05).

**Table 1:** Demographic characteristics of the sample.

Characteristics	Comorbidity group (*n* = 31)	Depression group (*n* = 26)	Healthy control group (*n* = 28)	*F*/*X* ^2^‐value	*p*‐Value
Age (years)	15.03 ± 1.33	14.85 ± 1.54	14.29 ± 1.56	2.005	0.141
Gender (female/male)	7/24	9/17	13/15	3.727	0.155
Level of education (years)	9.55 ± 1.34	9.46 ± 1.73	9.11 ± 1.73	0.616	0.543
HAMD	32.77 ± 9.48^#^	27.46 ± 7.53^#^	3.25 ± 1.49	41.677	<0.001 ^∗^
VAS	6.81 ± 0.95	0	0	—	—

*Note:* Numbers are expressed as cases or mean ± standard deviation.

Abbreviations: HAMD, hamilton depression scale; VAS, visual analog scale for pain.

^∗^Analysis of variance between groups *p*  < 0.05.

^#^There was no statistically significant difference in HAMD scores between the two groups.

### 3.2. Comparison of ALFF Among the Comorbidity Group, Depression Group, and Healthy Control Group

**Figure 2:**
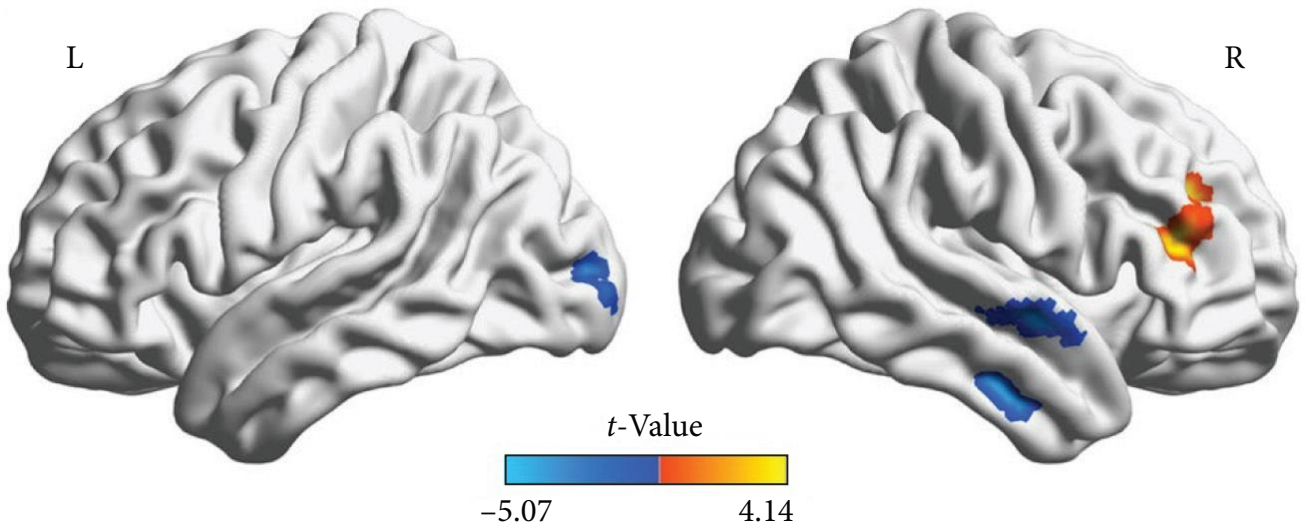
Compared with the depression group, the comorbidity group had increased ALFF in the right amygdala and right middle frontal gyrus (warm color) and decreased ALFF in the left middle occipital gyrus, right inferior temporal gyrus, and right superior temporal gyrus (cold color).

**Figure 3:**
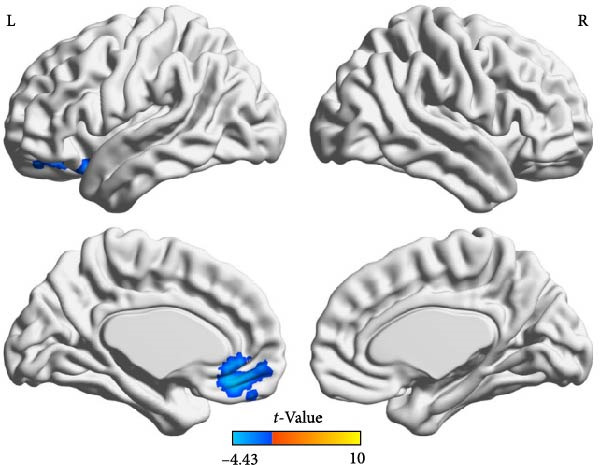
Decreased ALFF in the left rectus gyrus and left orbital inferior frontal gyrus in the comorbidity group compared with the healthy control group (cold color).

**Figure 4:**
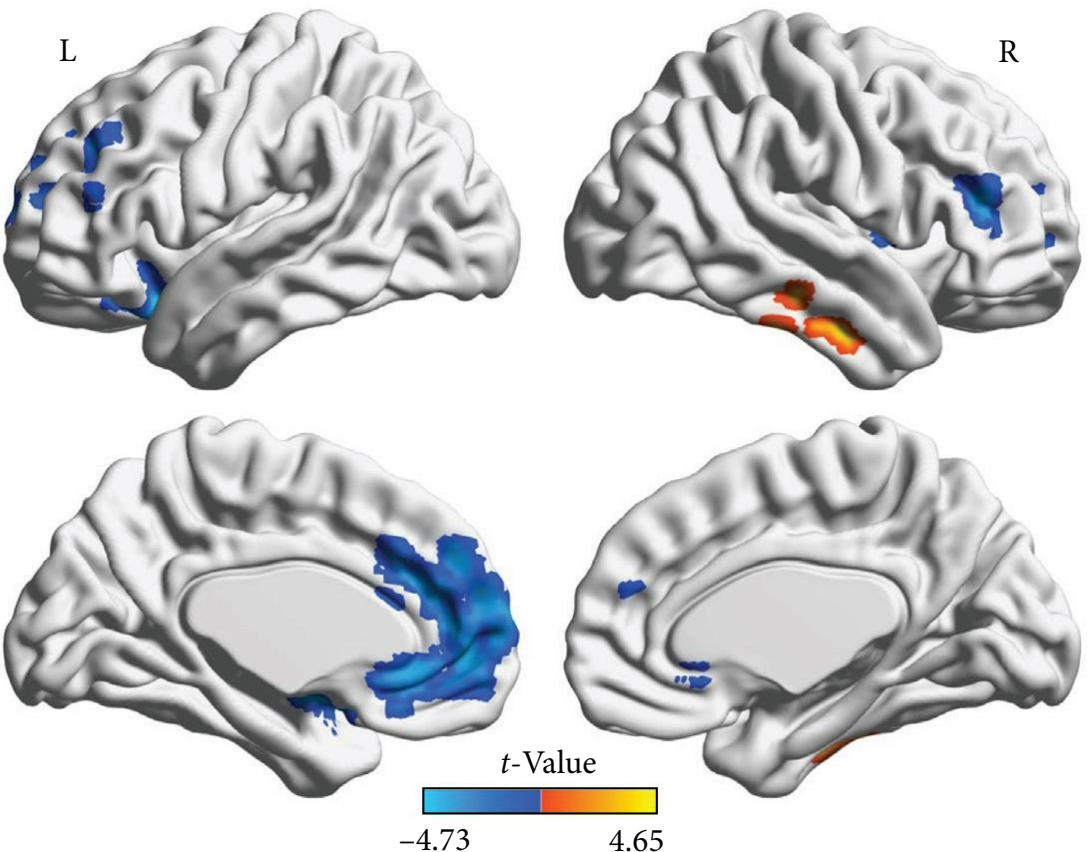
ALFF values were increased in the right inferior temporal gyrus in the depression group compared with the healthy control group (warm color). ALFF values were decreased in the left orbital inferior frontal gyrus, left medial superior frontal gyrus, bilateral middle frontal gyrus, right insular lobe, and right dorsal‐lateral superior frontal gyrus (cold color).

Compared to the depression group, the comorbidity group showed increased ALFF values in the right amygdala and right middle frontal gyrus, and decreased ALFF values in the left middle occipital gyrus, right inferior temporal gyrus, and right superior temporal gyrus (Table 2 and Figure 2). Compared to the healthy control group, the comorbidity group showed decreased ALFF values in the left gyrus rectus and left orbital inferior frontal gyrus (Table 3 and Figure 3). Compared to the healthy control group, the depression group showed increased ALFF values in the right inferior temporal gyrus, and decreased ALFF values in the left orbital inferior frontal gyrus, left medial superior frontal gyrus, bilateral middle frontal gyrus, right insula, and right dorsolateral superior frontal gyrus (Table 4 and Figure 4). All results were corrected using GRF, *p* < 0.05, and age, gender, education level, and motion parameters were used as covariates.

**Table 2:** Brain regions with differences in ALFF between comorbidity and depression groups.

Brain region	Voxels, *n*	MNI coordinates, mm (*x*, *y*, *z*)			Peak *t*‐values
Right amygdala	30	18	0	−18	3.9123
Right middle frontal gyrus	49	42	36	12	4.1399
Left middle occipital gyrus	31	−18	−96	6	−3.6563
Right inferior temporal gyrus	45	51	−12	−30	−5.0707
Right superior temporal gyrus	45	54	0	−9	−4.295

*Note:* GRF correction, voxel‐level *p*  < 0.005, cluster‐level *p*  < 0.05.

**Table 3:** Brain regions with differences in ALFF between comorbidity and healthy control groups.

Brain region	Voxels, *n*	MNI coordinates, mm (*x*, *y*, *z*)			Peak *t*‐values
Left gyrus rectus	105	−6	33	−12	−4.4289
Left orbital inferior frontal gyrus	74	−24	48	−12	−3.8269

*Note:* GRF correction, voxel‐level *p*  < 0.005, cluster‐level *p*  < 0.05.

**Table 4:** Brain regions with differences in ALFF between depression and healthy control groups.

Brain region	Voxels, *n*	MNI coordinates, mm (*x*, *y*, *z*)			Peak *t*‐values
Right inferior temporal gyrus	89	54	−9	−30	4.6466
Right middle frontal gyrus	48	48	36	15	−4.2049
Left middle frontal gyrus	65	−30	39	18	−4.3037
Right insula	43	36	6	9	−4.2858
Left medial superior frontal gyrus	499	−12	48	3	−4.7317
Left orbital inferior frontal gyrus	162	−24	0	−12	−4.5898
Right dorsolateral superior frontal gyrus	31	18	54	21	−4.0066

*Note:* GRF correction, voxel‐level *p*  < 0.005, cluster‐level *p*  < 0.05.

### 3.3. Comparison of ReHo Among the Comorbidity Group, Depression Group, and Healthy Control Group

**Figure 5:**
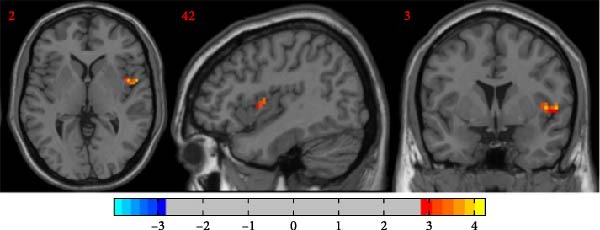
Increased ReHo in the right insula in the comorbidity group compared with the depression group (warm color).

**Figure 6:**
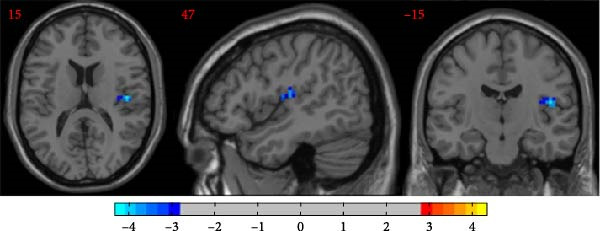
Decreased ReHo of the right Rolandic operculum in depressed patients compared with healthy controls (cold color).

Compared to the depression group, the comorbidity group showed increased ReHo values in the right insula (Figure 5). Compared to the healthy control group, the depression group showed decreased ReHo values in the right Rolandic operculum (Figure 6). All results were corrected using GRF, *p*  < 0.05, and age, gender, education level, and motion parameters were used as covariates.

### 3.4. Whole Brain Functional Connectivity Analysis

Using brain regions with ALFF differences between the comorbidity group and the depression group as seed points, whole‐brain functional connectivity analysis was performed. After DPABI variance analysis and post‐hoc GRF correction (voxel‐level *p*  < 0.005, cluster‐level *p*  < 0.05), with age, gender, education level, and motion parameters as covariates, the comorbidity group showed higher functional connectivity strength between the right inferior temporal gyrus and the right angular gyrus compared to the depression group, and higher functional connectivity strength between the left middle occipital gyrus and the right postcentral gyrus compared to the healthy control group. The functional connectivity strength between the left middle occipital gyrus and the left middle frontal gyrus was lower in the comorbidity group compared to the healthy control group. The depression group showed lower functional connectivity strength between the left middle occipital gyrus and the right lingual gyrus compared to the healthy control group, and higher functional connectivity strength between the right superior temporal gyrus and the right insula compared to the healthy control group (Table 5 and Figure 7). There were no significant differences in functional connectivity analysis using the right amygdala and right middle frontal gyrus as seed points among the three groups.

Figure 7Differences in functional connectivity among comorbid, depressed, and healthy controls. (A) decreased functional connectivity, (B) increased functional connectivity. MFG.L, left middle frontal gyrus; MOG.L, left middle occipital gyrus; LING.R, right lingual gyrus; PoCG.R, right postcentral gyrus; ANG.R, right angular gyrus; ITG.R, right inferior temporal gyrus; STG.R, right superior temporal gyrus; INS.R, right insula.

**Figure:**
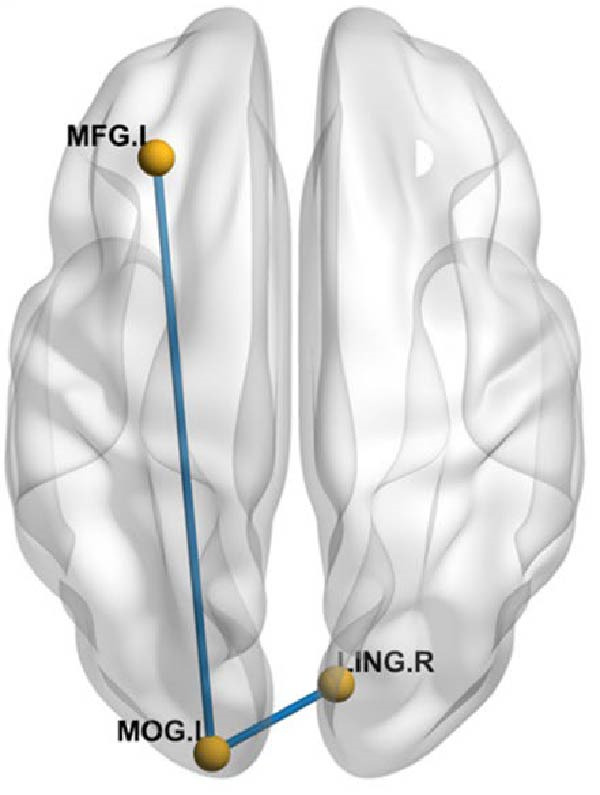
(A)

**Figure:**
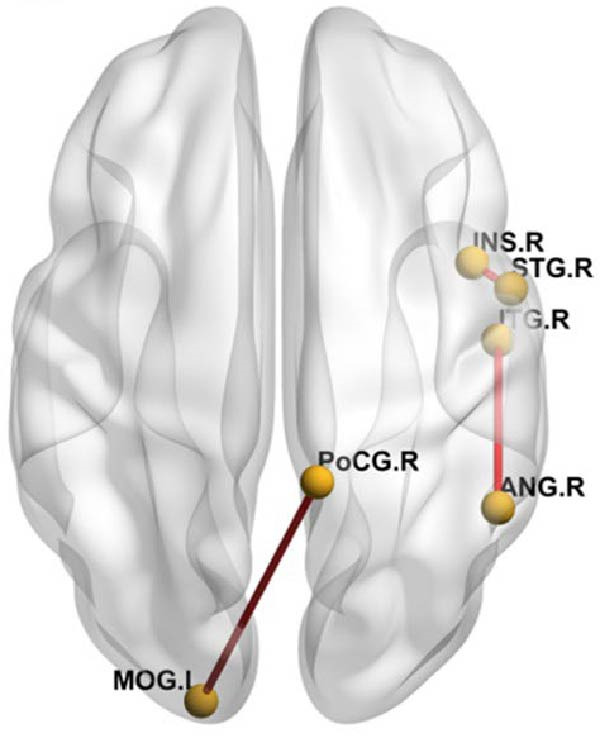
(B)

**Table 5:** Results of whole‐brain functional connectivity analysis.

Seed region	Brain region	Voxels, *n*	MNI coordinates, mm (*x*, *y*, *z*)			Peak *t*‐values
Left middle occipital gyrus	Right postcentral gyrus	27	9	−45	72	3.8007
	Left middle frontal gyrus	29	−30	33	42	−4.6111
	Right lingual gyrus	74	9	−81	0	−3.7696
Right superior temporal gyrus	Right insula	30	45	6	−15	3.3396
Right inferior temporal gyrus	Right angular gyrus	28	51	−51	33	3.5955

*Note:* GRF correction, voxel‐level *p*  < 0.005, cluster‐level *p*  < 0.05.

### 3.5. Correlation Analysis

**Figure 8:**
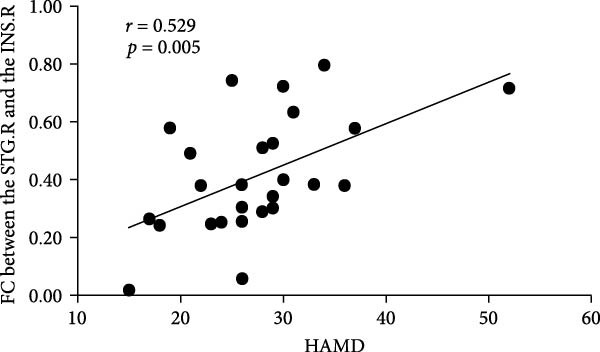
Functional connectivity values between the right superior temporal gyrus and the right insula were positively correlated with HAMD scores in adolescents with depression without comorbid pain.

Pearson correlation analysis was performed between ALFF, ReHo, and functional connectivity values of brain regions with differences among the comorbidity group, depression group, and healthy control group, and age, HAMD, and VAS scores. The results showed a positive correlation between the functional connectivity value of the right superior temporal gyrus and the right insula and HAMD scores in the depression group (*r* = 0.529, *p* = 0.005) (Figure 8). There were no significant correlations between ALFF, ReHo, functional connectivity values, and age, HAMD, and VAS scores in other brain regions, *p*  < 0.05.

## 4. Discussion

Based on the rs‐fMRI data, this study used multiple data analysis methods to find changes in ALFF, ReHo values, and functional connectivity strength in multiple brain regions in adolescent patients with comorbid pain and depression, and in depression patients. Compared to adolescent depression patients, adolescent patients with comorbid pain and depression showed abnormal local brain functional activities and functional connectivity in the right middle frontal gyrus, amygdala, inferior temporal gyrus, superior temporal gyrus, insula, and left middle occipital gyrus. Compared to healthy volunteers, adolescent depression patients showed brain functional abnormalities mainly in the prefrontal cortex, insula, and inferior temporal gyrus. These results reveal that there are differences and overlaps in the changes of neuronal activity, functional connectivity between the comorbidity group and depression group, indicating that there may be interactions between the neural activities of brain regions related to pain perception and emotion processing in adolescents with comorbid pain and depression.

### 4.1. Changes in Local Brain Functional Activities in Comorbidity Group and Depression Group

Previous studies on brain fMRI and transcranial magnetic stimulation (TMS) in patients with depression and pain have found partial overlaps and differences in their targets [20, 21]. Understanding the relationship between pain and depression, and clarifying the neural mechanisms of how pain symptoms affect brain functional changes in depression patients, can help the treatment and prognosis of comorbid patients.

This study compared brain functional activity changes in comorbid patients and depression patients, finding abnormal spontaneous neuronal activities in multiple brain regions in comorbid patients, suggesting dysfunction in pain perception and emotional regulation pathways in comorbid patients during pain processing. Specifically, decreased activation in the right inferior temporal gyrus, right superior temporal gyrus, and left middle occipital gyrus indicates impaired or inhibited function in these brain regions during pain processing in depression patients. Increased activation in the right middle frontal gyrus, right amygdala, and increased local consistency in the right insula suggest that these brain regions may be responsible for pain processing and regulation in depression patients and play a key role in connecting emotions and pain.

This study found that depressed adolescents had higher activation in the right superior temporal gyrus and lower activation in the left orbital inferior frontal gyrus, left medial superior frontal gyrus, bilateral middle frontal gyrus, left dorsolateral superior frontal gyrus, and right insula than healthy volunteers. These regions are the key brain regions of emotional regulation disorder in depressed patients, and partially overlap with the brain regions with abnormal brain structure in depression [22]. Decreased prefrontal cortex function in depression patients is a relatively consistent finding in depression research [23], suggesting that decreased ALFF values in the prefrontal cortex may be a manifestation of functional decompensation.

In this study, brain regions involved in emotional regulation in the depression group and brain regions involved in pain sensation processing in the comorbid group overlap in the right middle frontal gyrus, right inferior temporal gyrus, and right insula, consistent with previous research results [18, 24], further supporting the overlap between brain regions involved in emotional regulation and pain sensation pathways.

The ALFF value in the right middle frontal gyrus was lower in the depression group than in the healthy control group, but higher in the comorbid group than in the depression group, suggesting that decreased activation in the right middle frontal gyrus may be related to characteristic emotions in depression patients, while increased activation in the right middle frontal gyrus may play an important role in the occurrence of pain in depression patients. The middle frontal gyrus is an important part of the dorsolateral prefrontal cortex and a key node in the limbic neural circuit, involved in higher cognitive regulation, working memory, decision‐making, and emotional integration, playing an important regulatory role in emotional management [25, 26], and also participating in the descending pain regulation system by regulating cortical and subcortical injury pathways during pain processing [27]. Studies on tension‐type headache patients and adolescents with idiopathic musculoskeletal pain using MRI have shown decreased activation in the middle frontal gyrus [28, 29]. In contrast, patients with depression exhibit increased activation in the middle frontal gyrus during pain processing, which is speculated to be a compensatory response. Depression is commonly associated with dysfunction of monoamine neurotransmitters such as 5‐HT and norepinephrine. These neurotransmitters not only regulate mood but also participate in descending pain modulation pathways, inhibiting the transmission of nociceptive signals in the spinal dorsal horn. Depression leads to a reduction in these neurotransmitters, weakening descending inhibition and thereby enhancing pain perception [30]. Additionally, both depression and the unique neuroplastic changes during adolescence, such as synaptic pruning [31], contribute to impaired information transmission and integration between brain regions. These factors collectively lead to dysfunction in the descending pain modulation system. As the dorsolateral prefrontal cortex serves as a high‐level control center that initiates descending inhibitory commands, it exhibits compensatory hyperactivation when the descending inhibitory system is impaired.

The temporal lobe is not only involved in language and auditory‐visual information processing but also in the recall of personal experiences, playing an important regulatory role in emotions and memory. The ALFF value in the right inferior temporal gyrus was higher in adolescent depression patients than in healthy volunteers, but lower in adolescent comorbid patients than in depression patients. The right inferior temporal gyrus, superior temporal gyrus, and left middle occipital gyrus showed decreased activation during pain processing in depression patients. The inferior temporal gyrus and middle occipital gyrus are important nodes in the ventral visual pathway, and the superior temporal gyrus is part of the visual network. Although they are not part of the typical pain network, studies have shown that brain regions related to the visual and auditory systems are also involved in the pathophysiology of chronic pain [32, 33], but the specific mechanisms are unclear. It may be through cross‐modal integration in the central nervous system to participate in the functional integration of chronic pain [34].

The insula is part of the paralimbic cortex and has extensive fiber connections with other cortical and subcortical structures, integrating functional information from different systems such as sensation, emotion, motivation, and cognition, playing an important role in pain perception, emotional control, cognitive regulation, and sensorimotor functions. This study found increased ReHo values in the right insula in the comorbid group compared to the depression group, indicating enhanced local functional connectivity in the insula. Pain activation is mainly located in the posterior insula and adjacent dorsal midbrain, and its increased activity helps in pain perception control [35, 36]. Previous studies have shown decreased ReHo values in the insula in depression patients compared to healthy volunteers [37], but in this study, there was no significant difference in ReHo values in the insula between the depressed group and the healthy control group, while the ALFF values in the right insula of the depressed group were decreased, which was consistent with the results of Hu et al. [38]. This inconsistency may reflect developmental differences. The prefrontal‐limbic system circuit is still in the immature stage of development during adolescence.

Recent neuroimaging studies have shown that the amygdala is a key node in pain signal transmission and emotion generation, and its increased functional activation is one of the mechanisms of comorbid pain and depression [39, 40]. The amygdala is an important part of the limbic system and a core target for emotional regulation, subject to top‐down inhibitory regulation by the prefrontal cortex [41]. Increased amygdala activation in comorbid patients suggests abnormal emotional regulation function in depression patients, possibly related to decreased prefrontal cortex activity and reduced inhibition of the amygdala. This study did not find functional changes in the hippocampus, thalamus, or cingulate gyrus in adolescent comorbid patients, which are brain regions frequently studied in comorbid patients. This may be due to differences in methods and equipment parameters, or it may be related to the study population being first‐episode adolescent depression patients with incomplete brain development and relatively short disease duration, leading to less significant functional changes.

### 4.2. Changes in Whole‐Brain Functional Connectivity in Comorbidity Group and Depression Group

This study used five brain regions with ALFF differences between adolescent patients with comorbid pain and depression and depression patients as seed points for whole‐brain functional connectivity analysis. The results showed increased functional connectivity between the right inferior temporal gyrus and the right angular gyrus in the comorbid group compared to the depression group. Compared to the healthy control group, the comorbid group showed increased functional connectivity between the left middle occipital gyrus and the right postcentral gyrus. Compared to the healthy control group, the depression group showed decreased functional connectivity between the left middle occipital gyrus and the right lingual gyrus.

The increased functional connectivity between the right inferior temporal gyrus and the right angular gyrus in the pain comorbidity group suggests enhanced functional synergy between these brain regions. The inferior temporal gyrus is a key node of the ventral visual pathway, primarily associated with object recognition. The angular gyrus is a higher‐order multisensory integration hub and also serves as the visual language center (reading center). Our study found that the ALFF in the right inferior temporal gyrus was lower in the comorbidity group compared to the depression group, indicating that the increased functional synergy between the right inferior temporal gyrus and the angular gyrus may represent a compensatory response to the reduced functionality of the inferior temporal gyrus. This finding also suggests potential visual processing abnormalities in patients with comorbidity, though further task‐based studies are needed to substantiate this hypothesis.

The middle occipital gyrus and postcentral gyrus are part of the primary visual cortex and sensory cortex, respectively. The postcentral gyrus is part of the pain matrix, closely related to emotional processing, pain perception, and pain intensity recognition [42, 43]. As previously mentioned, the middle frontal gyrus is a key component of the dorsolateral prefrontal cortex and plays a pivotal role in emotion regulation and pain modulation. Abnormal functional connectivity in the middle occipital gyrus suggests impaired functional integration within the corresponding neural pathways in adolescent patients with comorbid pain. Previous studies have shown increased activation [10] or ReHo values [44] in the postcentral gyrus in comorbid patients compared to depression patients without pain, suggesting that functional changes in the postcentral gyrus play an important role in emotional processing of pain. Studies have found increased functional connectivity between the primary visual cortex and primary somatosensory cortex in patients with chronic low back pain [34], and Pujol et al. [33] found abnormal functional connectivity involving the visual and auditory cortex in patients with fibromyalgia, suggesting that this increased functional connectivity may be due to adaptive neural remodeling in the brain after repeated pain stimulation through cross‐modal mechanisms [34]. Therefore, it is speculated that increased functional connectivity between the left middle occipital gyrus and the right postcentral gyrus in the comorbid group may be due to neural remodeling caused by pain stimulation. However, no alterations in the functional activity of the postcentral gyrus were observed in our study, which may be related to the adolescent patient population included in the research. Factors such as ongoing brain development, relatively short disease duration, and consequently less pronounced functional changes could contribute to this finding.

Both patients with depression and those with chronic pain exhibit attentional bias, meaning they are more prone to focus on negative stimuli (such as pain sensations and sad memories). The reduced functional connectivity between the middle occipital gyrus and the middle frontal gyrus may suggest a dysfunction in the neural pathway responsible for integrating visual information in the middle occipital gyrus and subsequently conducting cognitive evaluation and emotional regulation. As a result, negative emotions cannot be effectively suppressed. This specific neural pathway may serve as an objective neuroimaging marker for patients with pain comorbidity. However, this is an inference based on current findings, and further longitudinal tracking or animal experiments are needed to confirm it.

The middle occipital gyrus and lingual gyrus are important nodes in the visual network, mainly responsible for visual information collection and processing. Neuroimaging studies have reported structural and functional abnormalities in the visual cortex of depression patients, manifested as increased or decreased volume and thickness in the occipital visual cortex [45, 46], and enhanced internal functional connectivity in the ventral/dorsal visual network [47]. Therefore, abnormal activity in the visual system is considered an important neuropathological basis for depression patients, and it is speculated that the visual cortex may play an important role in emotional regulation and integration in depression patients. However, depression is a heterogeneous disease, and the author did not find relevant studies on adolescent depression patients. The relationship between visual network dysfunction and emotional regulation needs further clarification.

In summary, pain processing in adolescent depression patients is related to changes in local neuronal activity and information integration and regulation disorders between neurons in brain regions such as the prefrontal cortex, limbic system, and temporo‐parieto‐occipital lobes, which may be the cause of emotional regulation and pain perception abnormalities in adolescent depression patients. Both adolescent comorbid patients and depression patients show visual cortex functional abnormalities, and it is speculated that their functional enhancement or reduction may be related to neural remodeling, functional compensation, and disease heterogeneity. The specific mechanisms need further clarification. These findings may provide potential biomarkers and therapeutic targets for future clinical interventions (such as TMS) and prognosis. Specifically, modulating the activity of key brain regions or aberrant functional connections could potentially alleviate comorbid symptoms more effectively.

## 5. Limitations

This study has the following limitations: (1) The study did not include a pure pain group or subgroup by different pain subtypes, which may introduce bias. (2) The sample size is small, which may affect statistical power. In the future, we will further expand the sample size and refine different subtypes to exclude sample heterogeneity and improve the stability of the results.

## 6. Conclusion

Compared to adolescent depression patients without pain, adolescent patients with comorbid pain and depression show differences in neuronal activity and functional connectivity in brain regions such as the middle frontal gyrus, amygdala, insula, and temporo‐parieto‐occipital lobes, indicating that abnormal neuronal activity in these brain regions may be the neural basis of comorbid pain and depression.

## Conflicts of Interest

The authors declare no conflicts of interest.

## Funding

This study was supported by the Science and Technology Program of Guizhou Province (Qian Ke He Basic‐ZK[2024] General 317) and the Science and Technology Program of Zunyi, Guizhou (Zun Shi Ke He HZ[2023] 263).

## Data Availability

The data that support the findings of this study are available from the corresponding author upon reasonable request.
